# The efficacy and safety of ketamine in the treatment of super-refractory status epilepticus: a systematic review

**DOI:** 10.1007/s00415-024-12453-7

**Published:** 2024-05-23

**Authors:** Mingyuan Yan, Tianye Sun, Jinmin Liu, Qing Chang

**Affiliations:** 1 Beijing University of Chinese Medicine, Beijing, China; 2 Dongfang Hospital, Beijing University of Chinese Medicine, No.6 Fangxingyuan Fengtai District, Beijing, 100078 China; 3 Dongzhimen Hospital, Beijing University of Chinese Medicine, Beijing, China

**Keywords:** Super-refractory status epilepticus, Status epilepticus, Ketamine, Epilepsy, Anesthetics

## Abstract

**Background:**

Ketamine, as an anesthetic, has been considered for terminating status epilepticus (SE); however, due to the urgency and severity of the condition, there are currently no randomized controlled trials internationally assessing the efficacy of ketamine for treating super-refractory status epilepticus. Similarly, there appears to be a lack of systematic reviews addressing this topic in the literature. Therefore, this systematic review aims to explore the effectiveness and safety of ketamine for terminating super-refractory status epilepticus.

**Methods:**

We conducted a systematic search on PubMed, EMBASE, and Web of Science databases. Manuscripts unrelated to the research on super-refractory status epilepticus were excluded, as were manuscripts published in non-English languages. The quality assessment and risk of bias were evaluated using the MINORS criteria. Data extraction was limited to qualitative synthesis due to the unsuitability of the data for meta-analysis.

**Results:**

Out of 782 studies retrieved from electronic databases, 11 met the inclusion criteria. Among them, 10 studies were retrospective, and 1 study was prospective. Patient data for inclusion were sourced from the case registries of the researchers' respective hospitals. Across all included studies, the administration of ketamine significantly reduced the duration of status epilepticus and demonstrated higher safety compared to patients not receiving ketamine treatment for super-refractory status epilepticus. Additionally, early administration of ketamine correlated with improved treatment outcomes. The risk of bias across all studies was deemed low.

**Conclusion:**

This systematic review suggests that ketamine may be a feasible treatment option for super-refractory status epilepticus. However, given the critical nature of super-refractory status epilepticus, clinicians should prioritize its termination over evaluating the efficacy of specific medications, ensuring patient safety remains paramount. If feasible in real-world medical settings, future research should focus on designing randomized controlled trials to observe the specific efficacy and mechanisms of ketamine. Careful validation is necessary before considering ketamine as a first-line treatment for super-refractory status epilepticus.

**Supplementary Information:**

The online version contains supplementary material available at 10.1007/s00415-024-12453-7.

## Introduction

Status epilepticus (SE) is characterized by continuous seizures with incomplete recovery of consciousness between episodes or seizures lasting for more than 30 min without self-termination. Refractory status epilepticus (RSE) is defined as seizures that persist despite appropriate doses of antiepileptic drugs (AEDs). Super-refractory status epilepticus (SRSE) is defined as status epilepticus that continues or recurs for 24 h or longer despite the use of anesthetics [1].

Unfortunately, RSE has a high likelihood of progressing to SRSE, which carries a very high mortality rate, approaching 50%. This places a significant burden on patients' families and society [2]. Currently, there is a lack of high-quality evidence supporting standardized treatment protocols for SRSE. Treatment in the intensive care unit (ICU) for SRSE lacks published guidelines, with most evidence derived from case reports or small case series.

The etiology of SRSE is complex and highly associated with changes in receptor characteristics of N-methyl-D-aspartate (NMDA) and gamma-aminobutyric acid (GABA) receptors [3]. Immediate administration of anesthetic agents is required once SE progresses to SRSE. Commonly used intravenous anesthetic anticonvulsant drugs include midazolam, propofol, thiopental sodium, and pentobarbital [4]. However, the efficacy of these drugs in terminating seizures is often suboptimal, sometimes requiring nearly a month to terminate SRSE [5]. In some cases, even with the use of multiple anesthetic agents, SRSE cannot be terminated, hence the potential role of ketamine in increasing the hope of seizure termination.

Due to the challenging nature of SRSE and the lack of high-level evidence or guidelines to guide treatment, clinicians must use all available means to terminate SRSE. Multiple case reports have highlighted the potential efficacy of ketamine. Therefore, this systematic review aims to provide evidence-based medicine evidence for the efficacy and safety of ketamine.

## Materials and methods

The guidelines outlined in the Preferred Reporting Items for Systematic Reviews and Meta-Analyses (PRISMA) were followed throughout this investigation [6]. The research protocol was submitted to PROSPERO for registration (registration number: CRD42024523938).

### PICO definition

In the current study, the Populations, Intervention, Comparison and Outcome (PICO) framework was defined as follows: P (Population): patients with supe-refractory status epilepticus; I (Intervention): Intravenous infusion of ketamine after ineffective infusion of one or more intravenous anesthetics; C (Comparator): Intravenous infusion of one or more intravenous anesthetics; O (Outcome): Remission rate of SRSE, adverse effects.

### Research question

Can ketamine, as a non first-line anesthetic, be effective and have high safety when other anesthetics are ineffective in treating SRSE?

### Data sources

A thorough literature search of electronic databases was performed, including PubMed, EMBASE, and Web of Science, the specific search terms for the three databases are as follows: Ketamine, Ketalar, Ketaset, Ketanest, Calipsol, Kalipsol, Calypsol, Ketamine Hydrochloride, refractory status epilepticus, super-refractory status epilepticus. Detailed search strategies can be found in the supplementary materials 1.

### Inclusion and exclusion criteria

Inclusion Criteria: (1) Studies focused on super-refractory status epilepticus; (2) Utilization of ketamine; (3) Published in English language. Exclusion Criteria: (1) Reviews, systematic reviews, meta-analyses, case reports, conference reports, animal experiments; (2) Inability to access the full text; (3) Non-English literature.

### Study selection

The retrieved original studies were independently screened by two researchers (MY and TS) through reading titles and abstracts to exclude any studies not relevant to the theme of this systematic review. If there was any uncertainty regarding the relevance of a study, the full text was reviewed to determine its inclusion. After screening and reviewing all potentially relevant studies, both researchers read the full text and finalized the list of included original studies based on the inclusion and exclusion criteria. In case of disagreement between the two researchers regarding any study, it was referred to a third researcher (QC) for joint resolution and decision-making.

### Data extraction

Two independent researchers (MY and JL) conducted data extraction from all studies that met the screening criteria for this systematic review. The data extracted from each study are as follows: (1) First author; (2) Publication year; (3) Study type; (4) Total sample size; (5) Gender ratio; (6) Average age; (7) Mortality rate; (8) Concurrent anesthetics; (9) Ketamine initiation time; (10) Ketamine infusion rate; (11) Ketamine maintenance duration; (12) Response rate; (13) Adverse events.

### Quality assessment

Two researchers (TS and JL) conducted quality assessment of all included studies based on the MINORS (Methodological items for non-randomized studies) criteria for non-comparative studies [7]. The MINORS criteria consist of 8 itemss: (1) A clearly stated aim; (2) Inclusion of consecutive patients; (3) Prospective collection of data; (4) Endpoints appropriate to the aim of the study; (5) Unbiased assessment of the study endpoint; (6) Follow-up period appropriate to the aim of the study; (7) Loss to follow up less than 5%; (8) Prospective calculation of the study size. Each item is scored as follows: 0 (not reported), 1 (reported but inadequate), or 2 (reported and adequate). The maximum score achievable is 16 for non-comparative studies. In case of disagreement, the matter is discussed with a third researcher (QC) for resolution.

### Data synthesis

The data extracted from each eligible study were qualitatively synthesized in the text of the article. Since all included studies were non-randomized controlled trials without a control group, meta-analysis was not employed. Therefore, we systematically examined and reviewed the extracted data to present the results in narrative form, evaluating the efficacy and safety of ketamine in terminating SRSE. Additionally, we proposed directions for future clinical practice and research.

## Results

### Study selection

**Fig. 1:**
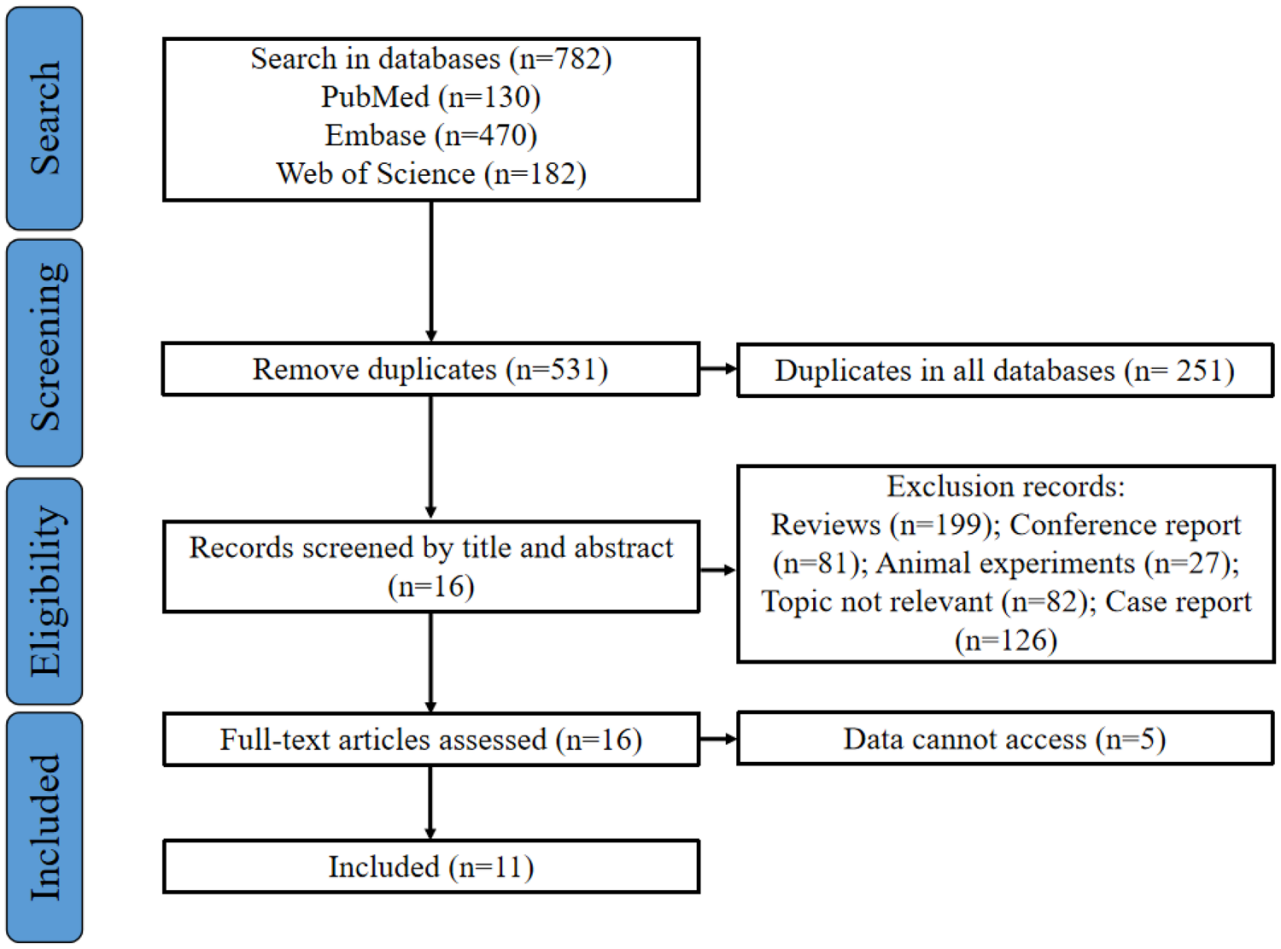
literature selection process

In PubMed, EMBASE, and Web of Science databases, a total of 782 records were retrieved. Among them, 251 were identified as duplicates. Following the screening of titles and abstracts of the remaining 531 records, 199 were excluded as reviews, 81 as conference reports, 27 as animal experiments, 126 as case reports, and 82 as irrelevant to the topic of this systematic review. Subsequently, full-text screening was attempted for the remaining 16 records, revealing that 5 records were inaccessible. Ultimately, the remaining 11 records met the selection criteria for this systematic review [8–18]. It is noteworthy that, regarding the study by Synowiec AS [18], while the title of the research pertains to RSE, a thorough examination of the entirety reveals its actual focus on SRSE, aligning with the definition of SRSE as seizures that persist or recur for 24 h or more despite the administration of anesthetics. Therefore, we also classify it as a study pertaining to SRSE. The literature selection process for this systematic review adhered to the PRISMA guidelines, as illustrated in Fig. 1.

### Study quality assessment

As all the included studies were non-randomized controlled trials, we utilized the MINORS criteria [7], designed for non-comparative studies, to assess their overall quality. This scoring tool lists 8 items for non-comparative studies. Upon careful examination by researchers, items 1–4 were reported in all studies, thus receiving a score of 2. However, since all 10 included studies were retrospective and blinding was not feasible, and the one prospective study did not report blinding assessment for outcome measures, they were all scored as 0 for blinding. For item 7, either the studies did not report loss to follow-up rates or the rates exceeded 5%, resulting in a score of 0 for this item as well. Items 6 and 8 met the criteria for a score of 2. Consequently, all studies were rated 12 points, indicating moderate quality. Specific scoring details are provided in Supplementary materials 2.

### Characteristics of included studies

The basic characteristics of the included eligible studies are presented in Table 1. Among the 11 studies, 10 were retrospective and 1 was prospective [10]. The publication years ranged from 2012 to 2024, with sample sizes varying from 7 to 68 cases. The patient population included infants, children, and adults, covering a wide age range, with a roughly balanced gender ratio. The duration of SRSE ranged from 1 to 96 days.

**Table 1:** Basic information included studies

References	Study type	Sample size	Gender(M/F)	Age	SRSE duration
[8]	Retrospective	68	22/46	53 ± 19 years	–
[9]	Retrospective	11	5/6	33–68 years	–
[10]	Prospective	11	6/5	48 ± 22 years	20 days (range 3–96)
[11]	Retrospective	7	4/3	44–86 years	–
[12]	Retrospective	43	22/20	67 years [Q1 59.3, Q3 72.0]	10 days (Q1 5.0, Q3 21.0)
[13]	Retrospective	38	–	91 days (8–340)	–
[14]	Retrospective	18	14/4	17 months [11 days − 24 years]	–
[15]	Retrospective	24	16/8	Responders 51 ± 14.8 years Non-responders 55.4 ± 20.5 years	Responders 10 days (3–21) Non-responders 10.4 days (3.5–23)
[16]	Retrospective	9	4/5	16 months to 10 years 5 months (5.2 ± 3.2)	6 days (mean 8.5 ± 7.5; range 2–26 days)
[17]	Retrospective	67	18/49	8–85 years (mean 58 years)	1–29 days (mean 6.0 days)
[18]	Retrospective	11	7/4	52 ± 18 years	7–38 days (mean 14.9 days)

### Ketamine intervention time point

All detailed study specifics are listed in Table 2. Among the 11 records, 9 reported detailed intervention time points following the occurrence of SRSE, generally ranging from 5 h to 26 days. Alkhachroum A [8] reported an average of 2 days (IQR 1–4.5) post-seizure onset; Basha M [9] ranged from 1 to 11 days, with an average of 5.4 days; Caranzano L [10] ranged from 2 to 20 days, with a median time of 4 days; Dericioglu N [11] initiated intervention between 4 and 19 days upon entry to the NICU; Höfler J [12] introduced ketamine with a median of 3 days (Q1 2.0, Q3 6.8); Kuki I [14] reported a median intervention time of 6.5 days (IQR 4.0–10.5); in Machado RA [15], ketamine intervention was divided into two groups: Responders: 1 day (range 0.5–2); Non-responders: 1 day (range 0.5–7); Rosati A [16] applied ketamine within a median time of 6 days after SRSE onset (range 5 h-26 days); although Sabharwal V [17] did not explicitly report time points, it was mentioned that most were between 24 and 48 h; Synowiec AS [18] reported ketamine intervention ranging from 1 to 11 days. Among these studies, there is no specific pattern to follow for the duration of ketamine intervention, which may be determined by the professional knowledge of doctors and the patient's condition at the time. However, in most studies, some patients started using ketamine within a day or even a few hours of the onset of SRSE.

**Table 2:** Details of utilization of ketamine among included studies

References	Patients and type	Katemine loading dosage	Katemine started time (days)	Katemine infusion rate (mg/kg/h)	Katemine maintained duration (days)	Respon-se rate	Morta-lity %	Adverse events
[8]	68 (all SRSE)	–	2 (IQR 1–4.5)	2.2 ± 1.8 (min–max 0.2–10)	2 (IQR 1–4)	43 (63.23%)	45.60	Not reported
[9]	11 (all SRSE)	–	0–11 (mean 5.4)	1–5 (mean 3.5)	2–27	4/11 (36.36%)	27.27	3 Pneumonia, 2 ICP (ICP, intracranial pressure); 3 sepsis, 2 cardiac failure, 2 hepatic failure, 1 renal failure
[10]	11 (all SRSE)	–	4 (range 2–20)	5 (range 2.5–15)	2 (range 1–16)	7/11 (63.63%)	36.30	Not reported
[11]	7 (6 SRSE)	0.5–2 mg/kg	4–19	1–5	3–24	5/7 (71.42%)	29.00	1 hepatic failure
[12]	42 (3 RSE, 39 SRSE)	–	3 (Q1 2.0, Q3 6.8)	2.55 (Q1 2.09, Q3 3.22)	4 (Q1 2.0, Q3 6.8)	27/42 (64.28%)	45.23	None
[13]	38 (35 RSE, 3 SRSE)	–	–	1–6	–	23/38 (60.52%)	44.73	1 hypertension
[14]	18 (all SRSE)	0.5–2 mg/kg	6.5 (IQR 4.0–10.5)	3.0 (IQR 2.0–4.3)	7.5 (IQR 2.8–15.5)	76% (IQR 53–94)	0.00	1 hypotension, 1 hypertension, 1 mild respiratory distress
[15]	24 (12 RSE, 12 SRSE)	–	Responders: 1 (range 0.5–2) Non-responders: 1 (range 0.5–7)	Responders: 2.2 (range 0.69–5) Non-responders: 2.4 (range 1–4)	–	12/24 (50%)	54.16	None
[16]	9 (1 RSE, 9 SRSE)	–	6 ( range 5 h–26 days)	40gamma/kg/min; (range 10–60)	6 (range 3–17)	6/9 (66.66%)	0.00	None
[17]	67 (all SRSE)	–	1–2	25–175 mcg/kg/min	6 (1–29)	91%	39.00	None
[18]	11 (all SRSE)	1–2 mg/kg	1–11 (mean 5)	0.45–2.1 (mean: 1.3)	4–28 (mean: 9.8)	100%	18.18	Not reported

### Ketamine infusion dose

Three studies [11, 14, 18] reported loading doses of ketamine ranging from 0.5 to 2 mg/kg administered prior to its use, with no adverse reactions documented. The issue of loading doses was not addressed in the remaining studies. Ten records describe specific infusion rates of ketamine: Alkhachroum A [8] administered ketamine at an average rate of 2.2 mg/kg/h (range 0.2–10); Basha M [9] ranged from 1–5 mg/kg/h (mean 3.5 mg/kg/h); Caranzano L [10] had a median dose of 5 mg/kg/h (range 2.5–15); Dericioglu N [11] ranged from 1 to 5 mg/kg/h; Höfler J [12] had a median infusion rate of 2.39 mg/kg/h (Q1 1.52, Q3 3.02); Jacobwitz M [13] reported starting at 1 mg/kg/h with a maximum infusion rate of 6 mg/kg/h; Kuki I [14] had a median infusion rate of 3.0 mg/kg/h (IQR 2.0–4.3); Machado RA [15] infusion rates: Responders (0.69 mg/kg/h-5 mg/kg/h); Non-responders (1 mg/kg/h-6 mg/kg/h); in Rosati A [16], the median dose was 40 gamma (mg)/kg/min (range 10–60 gamma [mg]/kg/min); in Synowiec AS [18], infusion rates ranged from 0.45 mg/kg/h-2.1 mg/kg/h. Overall, ketamine infusion rates varied from 0.45 mg/kg/h to 15 mg/kg/h, with only Caranzano L [10] using an extremely high dose of 15 mg/kg/h; the rest of the studies averaged around 2–4 mg/kg/h.

### Ketamine infusion duration

Nine records described the duration of ketamine administration. In the study by Alkhachroum A [8], the median duration of ketamine infusion was 2 days (range 1–4 days); Basha M [9] reported ketamine maintenance duration ranging from 2 to 27 days; in Caranzano L [10], the median duration of ketamine maintenance was 2 days (range 1–16); Dericioglu N [11] reported a total infusion duration of 3—24 days; Höfler J [12] reported a median duration of 4 days (Q1 2.0, Q3 6.8); Kuki I [14] reported a median duration of 7.5 days (IQR 2.8–15.5); in Rosati A [16], the median duration was 6 days (range 3–17); Sabharwal V [17] reported a duration range of 1 to 28 days for combined ketamine and propofol use, with an average duration of 3.6 days; Synowiec AS [18] reported durations ranging from 4 to 28 days. Overall, ketamine duration varied from 1 to 28 days, with longer durations such as 15.5 days, 17 days, 27 days, 28 days being rare occurrences. Most patients were able to terminate SE status within approximately 1–6 days.

### Ketamine in combination with other medications

The majority of studies have reported concomitant use of intravenous anesthetics and oral anti-epileptic drugs with ketamine. Among these studies, the utilization rate of midazolam stands indisputably as the highest, underscoring its frontline status in SRSE treatment. Propofol follows with a relatively high utilization rate, whereas Thiopental and Pentobarbital exhibit the lowest utilization rates. Alkhachroum, A [8] reported midazolam infusion rates of 1 (0.9) mg/kg/h prior to ketamine initiation and 1 (0.81) [0.2–3] mg/kg/h post-ketamine initiation (mean (SD), [minimum – maximum]). Sabharwal et al. [17] documented propofol dosages ranging from 25 to 140 mg/kg. Other studies did not specify dosages of intravenous anesthetics used. As for oral medications, it is challenging to discern a pattern due to variability across studies. Commonly utilized drugs include levetiracetam, phenytoin, phenobarbital, lorazepam, and sodium valproate. Most of these studies reported concurrent administration of oral anti-epileptic or sedative medications alongside intravenous anesthetics. Refer to Table 3 for further details.

**Table 3:** Details of the use of anti-seizures medications and anesthetics

References	Sample size	Number of concurrent anesthetics	Concurrent anesthetics	Anti-seizures medications
[8]	68	2 ± 1	Midazolam (68, 100%); Pentobarbital (10, 14.7%); Propofol (10, 14.7%)	Levetiracetam 40% Phenytoin 25% Lacosamide 14% Valproic Acid 7% Clobazam 2% Phenobarbital 2% Gabapentin 2%
[9]	11	1–2	Midazolam (9, 81.8%); Pentobarbital (3, 27.3%); Propofol (3, 36.4%)	–
[10]	11	1–3	Midazolam (9, 81.8%); Thiopental (1, 9.1%); Propofol (2, 18.2%)	Clonazepam (1, 9.1%); Phenytoin (4, 36.4%); Levetiracetam (5, 45.5%); Topiramate (2, 18.2%); Carbamazepine (1, 9.1%); Pregabaline (2, 18.2%); Phenobarbital (4, 36.4%); Valproate (7, 63.6%); Lacosamide (5, 45.5%);
[11]	7	1–3	Midazolam (6, 85.7%); Propofol (2, 28.6%); Thiopental (1, 14.3%); One patient did not use any anesthesia	Levetiracetam (7, 100%); Clobazam (4, 57.1%); Topiramate (5, 71.4%); Oxcarbazepine(1, 14.3%); Lacosamide (5, 71.4%); Phenytoin (4, 57.1%)
[12]	42	1–4 (Q1 2.0, Q3 4.0)	Only 17 patients were mentioned to have used propofol, and information on the remaining anesthetics was not mentioned	–
[13]	38	–	–	–
[14]	18	–	–	–
[15]	24	1–3	Ketamine responders (n = 12) Midazolam (5, 41.67%); Propofol (3, 25%); Ketamine non-responders (n = 12) Midazolam (5, 58.3%); Propofol (6, 50%) Pentobarbital (3, 25%);	Ketamine responders (n = 12): Levetiracetam (8, 66.7%); Lacosamide (3,25%); Perampanel (3,25%); Brivaracetam (1,8.3%); Phenytoin (1, 8.3%); Topiramate (1, 8.3%); Valproic acid (1, 8.3%); Ketamine non-responders (n = 12): Levetiracetam (8,66.7%); Lacosamide (5, 41.7%); Valproic acid (4,33.3%); Perampanel (1, 8.3%) Phenytoin (1, 8.3%); Retigabine (1, 8.3%); Carbamazepine (1, 8.3%); Stiripentol (1, 8.3%)
[16]	9	1–2	Midazolam (9, 100%); Propofol (1, 11.1%)	Phenobarbital (8,88.9%); Topiramate (2, 22.2%) Rufinamide (1, 11.1%); Clonazepam (1, 11.1%); Stiripentol (1, 11.1%); Clobazam (1, 11.1%); Lorazepam (1, 11.1%); Nitrazepam (1, 11.1%); Felbamate (1, 11.1%); Valproate (1, 11.1%); Phenytoin (1, 11.1%)
[17]	67	–	Propofol (67, 100%);	–
[18]	11	1–3	Midazolam (1, 9.1%); Propofol (7, 63.6%); Pentobarbital (1, 9.1%)	Lorazepam (9, 81.8%); Phenytoin (7, 63.6%); Valproic acid (5, 45.5%); Phenobarbital (4, 36.4%); Carbamazepine (2, 18.2%); Gabapentin (1, 9.1%); Diazepam (1, 9.1%); Lamotrigine (1, 9.1%)

### Adverse effects

Three studies did not report adverse effects [8, 10, 18], while four studies did not mention adverse effects [12, 15–17]. Basha M [9] reported 3 cases of pneumonia, 2 cases of high intracranial pressure (ICP), 3 cases of sepsis, 2 cases of cardiac failure, 2 cases of hepatic failure, and 1 case of renal failure; Dericioglu et al. [11] reported 1 case of hepatic failure; Jacobwitz et al. [13] reported 1 case of hypertension; Kuki et al. [14] reported 1 case of hypotension, 1 case of hypertension, and 1 case of mild respiratory distress. The occurrence of more severe adverse effects may be related to the patients' underlying diseases or poorer overall conditions and cannot be directly attributed to ketamine. The remaining milder adverse effects were transient and temporary, resolving after treatment without recurrence. Additionally, although Basha et al. [9] reported 2 cases of high ICP, one individual already had high ICP before ketamine use, and the other developed it due to a cerebral hemorrhage.

### Overall analysis of the included studies

In the study by Alkhachroum et al. [8], the average Status Epilepticus Severity Score (STESS) remained around 4 points. Only this study reported seizure burden within 24 h, with 81% of patients experiencing at least a 50% reduction, and 63% of patients achieving seizure cessation within 24 h. Following cessation of ketamine, 79% of patients experienced at least a 50% reduction in seizure burden, and 65% of patients achieved seizure cessation. Prior to ketamine administration, patients received an average of two anesthetic treatments, such as midazolam, propofol, or pentobarbital. Furthermore, the study supports that even with high doses and prolonged administration of ketamine, there were no adverse effects ICP and cerebral blood flow (CBF), and it even improved cerebral hemodynamics. In the study by Basha et al. [9], the maximum number of anesthetics used prior to ketamine administration was two, which included midazolam, propofol, or pentobarbital. However, in this study, ketamine had the lowest response rate, at 36.36%. Following the initiation of ketamine infusion in this study, regardless of high or low doses, and whether used in combination with other anesthetics, a characteristic rhythmic electroencephalogram (EEG) pattern emerged throughout the course of ketamine use: generalized frontally predominant archiform theta to beta rhythms (7–20 Hz) of 25–35 μV in amplitude. This characteristic rhythm was termed “ketamine EEG effect” by the authors. Patients in this cohort did not achieve burst-suppression (BS) pattern, suggesting that achieving BS pattern may not always be necessary for treating RSE. The study also found that even with high doses and prolonged use, ketamine did not lead to any adverse events, including exacerbating underlying conditions. However, despite early intervention with ketamine in this cohort, early control of RSE was not achieved, contrary to the results of most studies, and the reasons for this phenomenon remain unclear. Nevertheless, a significant contribution of this study is the discovery of the “ketamine EEG effect,” which may serve as a potential biomarker guiding the use of ketamine in the future. In the study by Caranzano L [10], the average Status Epilepticus Severity Score (STESS) was around 3. The intervention time for ketamine ranged from 2 to 20 days, with a median time of 4 days. In this study, one patient had an astonishing duration of SE lasting for 96 days until ketamine intervention. The maximum dose in this study was also the highest, reaching 15 mg/kg/h. Ketamine use resulted in seizure cessation in 7 patients, but only 3 individuals (27%) achieved complete seizure cessation after discontinuing ketamine use and in 4 out of 11 patients, SRSE ceased during ketamine administration, only to recur upon cessation of ketamine treatment. In Dericioglu N [11]'s study, Glasgow Coma Scale scores ranged from 3 to 9. STESS scores were 4 or 5. Prior to ketamine infusion, midazolam, propofol, and sodium thiopental were used either alone or in combination with ketamine. All patients had poor prognosis with Modified Rankin Scale scores ranging from 4 to 6 at discharge, likely due to the patients’ underlying diseases. This study also suggested that earlier intervention with ketamine could lead to earlier termination of SRSE, as the only two non-responding patients received ketamine later than other responding cases (6–7 vs 2–4 days). This difference was statistically significant (P = 0.047). Additionally, there seemed to be a trend suggesting that longer duration of ketamine infusion might lead to better efficacy, although it did not reach statistical significance (P = 0.285). In Höfler et al. [12]’s study, 1–4 intravenous anesthetics were used before ketamine administration. Ketamine was introduced with a median of 3 days (Q1 2.0, Q3 6.8), and the median infusion rate was 2.39 mg/kg/h (Q1 1.52, Q3 3.02), which was lower than in other studies. The median duration of ketamine was 4 days (Q1 2.0, Q3 6.8). In this study, the response rate to ketamine was 64.28%, which, although relatively high, might be underestimated due to the smaller doses and shorter duration of ketamine use compared to other studies. Furthermore, no adverse reactions related to ketamine were observed in this study. In the study by Jacobwitz M [13], all patients who received ketamine were infants or children. Although the study did not report the start time and duration of ketamine, it reported a maximum infusion rate of 6 mg/kg/h. Ketamine was compared to midazolam in this report, with ketamine (61%; 23/38) terminating SE more often than midazolam (28%; 22/79) (P < 0.01). Additionally, 32 patients in this study received ketamine after midazolam failed, resulting in seizure termination in 32% and seizure reduction in 45%, with an overall efficacy of 77%. In this study, none of the 38 patients receiving ketamine had BS pattern on EEG, suggesting that achieving BS pattern may not always be necessary for treating SE. As for adverse reactions, the incidence rate of adverse reactions with midazolam was significantly higher than that with ketamine (24%; 20/79) vs (3%; 1/38), (P = 0.016). Only one case of hypertension was reported in the ketamine group. In Kuki I [14]'s study, patients ranged from infants to adults, and although the number of anesthetics used before ketamine was not mentioned, the study reported the use of 5 [IQR 3–5] intravenous antiepileptic drugs. Patients were divided into inflammatory and non-inflammatory etiology groups, with significantly longer ketamine treatment duration in the inflammatory group compared to the non-inflammatory group (p = 0.045). In both groups, median seizure frequency significantly decreased after ketamine administration (inflammatory: p = 0.0020; non-inflammatory: p = 0.0078). Additionally, the study reported significantly lower seizure frequency before ketamine administration in the non-inflammatory etiology group compared to the inflammatory group (p = 0.026). Neurological function was also significantly better in the non-inflammatory etiology group (p = 0.0003). This suggests that despite both being status epilepticus, non-inflammatory etiology may have a better prognosis, although the overall response rate to ketamine was 76%. Adverse reactions reported in this study included 1 case of hypotension, 1 case of hypertension, and 1 case of mild respiratory distress, all of which were mild and transient, disappearing after treatment. In Machado RA [15]'s study, 24 patients received ketamine treatment, and the study directly compared responders (n = 12) and non-responders (n = 12). It was found that in responders, patients began to show beta rhythms on EEG after ketamine use, which continued until seizure cessation, and this phenomenon was significantly higher in responders compared to non-responders (100% vs 33.3%), which could also be a characteristic manifestation of ketamine use. The study also did not report any adverse events. In Rosati et al. [16]’s study, the included patients were also children. Prior to ketamine use, at least one intravenous anesthetic was used, including midazolam, propofol, or sodium thiopental. Ketamine was administered within 6 days (range 5 h–26 days) for SE, with a median dose of 40 gamma (mg)/kg/min (range 10–60 gamma [mg]/kg/min), and a median duration of 6 days (range 3–17). None of the patients who received ketamine treatment experienced recurrent SE. Burst-suppression pattern on EEG was observed in 5 children. Ketamine did not lead to any adverse reactions. In Sabharwal et al. [17]’s study, patients ranged in age from 8 to 85 years old. Ketamine intervention was also relatively early, with most occurring within 24–48 h. Six patients were initiated on ketamine, while propofol was the initial medication for 61 patients. The duration of ketamine and propofol co-administration ranged from 1 to 28 days, with an average duration of 3.6 days. Although the study did not provide detailed descriptions of ketamine use, it reported that short- or long-term infusion of ketamine, with or without propofol infusion, effectively controlled SRSE. Ketamine, with its positive hemodynamic characteristics of aggressive fluid resuscitation, was beneficial for patients with RSE. The study did not report any adverse reactions. In Synowiec A [18]'s study, 11 patients were included, with 5 patients terminating seizures within 24 h, 3 within 72 h, and the remaining 3 requiring longer treatment but eventually achieving seizure cessation. The study reported that ketamine could stably improve patients' hemodynamics, and no adverse effects were found.

## Discussion

In the systematic review included in this study, the etiologies of SRSE include cardiac arrest, New-Onset Refractory Status Epilepticus (NORSE), intracerebral hemorrhage, brain tumors, hypoxic brain injury, cerebrovascular disease, central nervous system (CNS) infections, bacterial meningitis, viral encephalitis, ischemic heart disease, low antiepileptic drug levels, metabolic disturbances, and acute causes. These etiologies vary in their acuteness and severity, but all ultimately lead to the occurrence of SRSE, which can be very serious and even life-threatening. Due to the multifaceted nature of the causes leading to this condition, terminating such states can be quite challenging. Therefore, all included studies used intravenous anesthetics before ketamine but failed to terminate this state, requiring the combination or sequential use of multiple anesthetics to achieve termination. All studies used one or more intravenous anesthetics before ketamine, including midazolam, propofol, pentobarbital, and thiopental sodium. These drugs are mostly GABA_A_ receptor agonists and are closely associated with cardiopulmonary depression, hypotension, and other adverse effects. Therefore, it is common to administer vasopressors alongside these drugs, and prolonged use carries a significant risk of shock or even death for the patients. Animal experiments suggest that changes in GABA_A_ receptor transport may be one of the reasons for the failure of these drugs' treatment. As the duration of seizures lengthens, NMDA receptor antagonists may become relatively more effective, and the simultaneous use of GABA_A_ agonists and NMDA antagonists has synergistic effects. It has been reported that combining ketamine with benzodiazepines can enhance its efficacy, this perspective aligns with the studies included in our systematic review, demonstrating that the co-administration of midazolam, a benzodiazepine, with ketamine exhibited the highest utilization rate, with nearly all studies incorporating midazolam. However, whether ketamine in combination with intravenous anesthetics other than midazolam exhibits mutual enhancement effects cannot be conclusively determined solely through systematic review. Although it may be associated with receptors agonists or antagonists relevant to SRSE onset, this requires more rigorous animal and clinical trials for elucidation [19–23].

The present systematic review focuses solely on evaluating the efficacy and safety of ketamine in the treatment of SRSE, without providing specific descriptions of etiology or mechanisms. Across almost all included studies, there is a high response rate to ketamine intervention in SRSE, ranging from a maximum of 91% [17] to a minimum of 36.36% [9], with response rates in other studies also exceeding 50%. The timing of ketamine intervention varies among these studies, showing no consistent trend, likely influenced by clinicians’ knowledge and judgment as well as individual patient conditions. Early administration of ketamine is implied or explicitly stated to yield greater efficacy, while low doses may not terminate seizures. Although consistent with the findings of some studies, which suggest that ketamine administration starting on the 8th day after onset or at doses < 0.90 mg/kg/h may not control seizures [24], there is currently no high-level evidence to support this view, and it may be subject to selection bias. Nevertheless, based on the studies included in this systematic review, we still recommend early intervention with ketamine following detailed and cautious analysis of individual patient circumstances, particularly in patients with circulatory volume issues.Studies by Alkhachroum et al. [8], Sabharwal et al. [17], and Synowiec et al. [18] all indicate that ketamine can improve patient hemodynamics and reduce the need for vasopressor medications. Despite the administration of ketamine, these studies still reported a relatively high mortality rate. However, this is unlikely to be attributed to the infusion rate or duration of ketamine administration. The most probable reasons remain the etiology and severity of SRSE inherent to the patients themselves, consistent with the conclusions drawn in these reports. Furthermore, according to Kuki et al. [14], it appears that patients with non-inflammatory etiologies of SRSE have a better prognosis compared to those with inflammatory etiologies. However, due to the small sample size of this study, we cannot conclusively determine whether this theory holds true across all SRSE patient populations. More rigorous clinical trials are still needed to validate this perspective.

In summary, several key findings emerge from this systematic review: (1) Ketamine use may lead to the “ketamine EEG effect,” possibly representing a characteristic rhythm, but further experimentation is needed for confirmation; (2) Achieving burst suppression mode is not always necessary for treating SRSE; (3) The appearance of beta rhythms may also be indicative of successful ketamine use, but further clinical trials are needed for verification; (4) Patients across various age groups, from infants to the elderly, were covered in this systematic review, demonstrating ketamine’s efficacy in terminating SE across different age ranges; (5) Ketamine has advantages in improving hemodynamics compared to other intravenous anesthetics; (6) Even at high doses and prolonged infusion times, ketamine still exhibits fewer side effects and high safety, although this view is only supported by studies included in this systematic review and requires higher-level evidence for confirmation.

## Conclusion

Ketamine, when used after other intravenous anesthetics have proven ineffective, can terminate SRSE and possesses a relatively high level of safety. However, its administration should be determined by clinicians following a detailed assessment of the patient’s condition, and the dosage should not be too low. Nevertheless, due to the non-randomized nature of the studies included in this systematic review, the evidence level of the conclusions drawn is relatively low. To establish whether ketamine is the optimal choice, randomized controlled trials are needed. However, given the specificity and severity of SRSE, conducting such trials is challenging. The conclusions of this systematic review support the high efficacy and safety of ketamine for SRSE patients across all age groups. Therefore, following careful evaluation of the patient's condition, ketamine should be administered as early as possible.

### Supplementary Information

Below is the link to the electronic supplementary material.

Supplementary file1 (DOCX 11 KB)

Supplementary file2 (DOCX 14 KB)

Supplementary file3 (XLSX 11 KB)

Supplementary file4 (XLSX 12 KB)

## Data Availability

The original contributions presented in the study are included in the article, further inquiries can be directed to the corresponding author.
