# Supplementary material for: The efficacy and safety of ketamine in the treatment of super-refractory status epilepticus: a systematic review
Source: J Neurol. 2024 May 23;271(7):3942–52. doi: 10.1007/s00415-024-12453-7 (PMC11233303; doi:10.1007/s00415-024-12453-7)
Supplement: Supplementary file 1 — Supplementary file1 (DOCX 11 KB) [file 415_2024_12453_MOESM1_ESM.docx]

Search strategy

**Pubmed**

#1 "Ketamine"[MeSH Terms] 15403

#2 "Ketamine"[Title/Abstract] OR "Ketalar"[Title/Abstract] OR "Ketaset"[Title/Abstract] OR "Ketanest"[Title/Abstract] OR "Calipsol"[Title/Abstract] OR "Kalipsol"[Title/Abstract] OR "Calypsol"[Title/Abstract] OR "ketamine hydrochloride"[Title/Abstract] 23261

#3 #1 OR #2 24970

4# "refractory status epilepticus"[Title/Abstract] OR "super refractory status epilepticus"[Title/Abstract] 1422

#5 #3 AND #4130

**Embase**

#1. 'ketamine'/exp OR 'ketamine' OR 'ketalar'/exp OR 'ketalar' OR 'ketaset'/exp OR 'ketaset' OR 'ketanest'/exp OR 'ketanest' OR 'calipsol'/exp OR 'calipsol' OR 'kalipsol'/exp OR 'kalipsol' OR 'calypsolketamine hydrochloride' 67999

#2. 'refractory status epilepticus' OR 'super-refractory status epilepticus' 2493

#3. #1 AND #2 470

**Web of science**

TS=(Ketamine OR Ketalar OR Ketaset OR Ketanest OR Calipsol OR Kalipsol OR Calypsol OR Ketamine Hydrochloride) AND TS=(refractory status epilepticus OR super-refractory status epilepticus) 182
