# Supplementary material for: The efficacy and safety of ketamine in the treatment of super-refractory status epilepticus: a systematic review
Source: J Neurol. 2024 May 23;271(7):3942–52. doi: 10.1007/s00415-024-12453-7 (PMC11233303; doi:10.1007/s00415-024-12453-7)
Supplement: Supplementary file 2 — Supplementary file2 (DOCX 14 KB) [file 415_2024_12453_MOESM2_ESM.docx]

| **Quality assessment in MINORS** | | | | | | | | | |
| --- | --- | --- | --- | --- | --- | --- | --- | --- | --- |
| **Study** | **Methodological items for non-randomized studies (non-comparative studies)** | | | | | | | | |
|  | Item 1 | Item 2 | Item 3 | Item 4 | Item 5 | Item 6 | Item 7 | Item 8 | Total score |
| Alkhachroum, A 2020 | 2 | 2 | 2 | 2 | 0 | 2 | 0 | 2 | 12 |
| Basha, M M 2015 | 2 | 2 | 2 | 2 | 0 | 2 | 0 | 2 | 12 |
| Caranzano, L 2022 | 2 | 2 | 2 | 2 | 0 | 2 | 0 | 2 | 12 |
| Dericioglu, N 2020 | 2 | 2 | 2 | 2 | 0 | 2 | 0 | 2 | 12 |
| [Höfler](https://pubmed.ncbi.nlm.nih.gov/?size=50&term=H%C3%B6fler+J&cauthor_id=27465262), L 2016 | 2 | 2 | 2 | 2 | 0 | 2 | 0 | 2 | 12 |
| Jacobwitz, M 2023 | 2 | 2 | 2 | 2 | 0 | 2 | 0 | 2 | 12 |
| Kuki, I 2024 | 2 | 2 | 2 | 2 | 0 | 2 | 0 | 2 | 12 |
| Machaodo, R A 2022 | 2 | 2 | 2 | 2 | 0 | 2 | 0 | 2 | 12 |
| Rosati, A 2012 | 2 | 2 | 2 | 2 | 0 | 2 | 0 | 2 | 12 |
| Sabharwal, V 2015 | 2 | 2 | 2 | 2 | 0 | 2 | 0 | 2 | 12 |
| Synowiec, A S 2013 | 2 | 2 | 2 | 2 | 0 | 2 | 0 | 2 | 12 |

Item1: A clearly stated aim; Item2: Inclusion of consecutive patients; Item3: Prospective collection of data; Item4: Endpoints appropriate to the aim of the study; Item5: Unbiased assessment of the study endpoint; Item6: Follow-up period appropriate to the aim of the study; Item7: Loss to follow up less than 5%; Item8: Prospective calculation of the study size.

The items are scored 0 (not reported), 1 (reported but inadequate) or 2 (reported and adequate). The global ideal score being 16 for non-comparative studies.
